# Resuscitation after cardiac arrest in a septic porcine model: adding vasopressin vs epinephrine alone administration

**DOI:** 10.1186/1756-0500-7-492

**Published:** 2014-08-04

**Authors:** Thomas Loukas, Ioannis Vasileiadis, Helen Anastasiou, Eleftherios Karatzanos, Vasiliki Gerovasili, Emmeleia Nana, Giorgos Tzanis, Serafim Nanas

**Affiliations:** 1 First Critical Care Department, Evangelismos Hospital, National and Kapodistrian, University of Athens, Athens, Greece

**Keywords:** Cardiopulmonary resuscitation, Sepsis, Ventricular fibrillation, Cardiac arrest, Lipopolysaccharide

## Abstract

**Background:**

Vasopressin administration has been tested in cardiac arrest. However it has not been tested when cardiac arrest occurs in certain circumstances, as in sepsis, where it may have a major role. The aim of the study was to investigate survival after cardiac arrest in a septic porcine model compared with healthy animals and to explore the effectiveness of adding vasopressin vs epinephrine alone administration.

**Methods:**

Thirty five healthy piglets of both genders were studied. The piglets were randomly assigned into three groups: group A (n = 8), group B (n = 14), group C (n = 13). Animals of groups B and C were given endotoxin to mimic a septic state before arrest. We applied the same resuscitation protocol to all pigs but we replaced the first dose of epinephrine with vasopressin in pigs of group C. Following surgical preparation and 30 min resting period, baseline measurements were recorded. In order to assess tissue oxygenation, we implemented Near Infrared Spectroscopy (NIRS) with the vascular occlusion technique (VOT) in thirteen lipopolysaccharide (LPS)-treated animals, occluding abdominal aorta and inferior vena cava. Afterwards, LPS (100 μg/kg) was infused in a 30 min period to animals of groups B and C and normal saline to group A. New NIRS measurements were obtained again. Subsequently, we provoked ventricular fibrillation (VF). After 3 min of untreated VF, open chest cardiopulmonary resuscitation (CPR) was performed manually. Primary end point was the restoration of spontaneous circulation (ROSC).

**Results:**

The chance of ROSC for the groups A, B and C was 75%, 35.7%, and 30.7% respectively. A significant difference in ROSC was established between septic (group B + C) and non septic piglets (group A) (P = 0.046). Vasopressin administration had no effect in outcome. LPS administration decreased oxygen consumption rate, as assessed by NIRS, in peripheral tissues (22.6 ± 7.2. vs 18.5 ± 7.2, P = 0.07).

**Conclusion:**

Septic piglets have fewer chances to survive after cardiac arrest. No difference in outcome was observed when the first dose of epinephrine was replaced with vasopressin to treat cardiac arrest in the LPS-treated animals.

## Background

Sepsis is a major health problem worldwide, especially in Intensive Care Units (ICUs). Its incidence and the sepsis-related mortality are gradually increasing [1].

Sepsis syndrome adversely affects microcirculation [2] as well as mitochondrial respiration [3], compromising oxygen availability and utilization in tissues. In experimental animal studies, bacterial cell wall lipopolysaccharide (LPS) is used to induce endotoxemia and create a sepsis-like state. LPS infusion induces similarly microcirculatory alterations [4] and inhibits mitochondrial respiration and oxygen consumption rate even in the early stages after infusion [5].

Surviving sepsis campaign for management of severe sepsis and septic shock suggests that arginine-vasopressin (AVP, up to 0.03 U/min) can be added to norepinephrine (NE) in order to raise mean arterial pressure and decrease NE dosage [6]. Rationales for AVP use are mainly a relative deficiency of AVP in septic shock and improved hemodynamics, as AVP restores the vascular reactivity to catecholamines, which is reduced, and decreases catecholamines requirements [7,8].

A vasopressor during cardiopulmonary resuscitation (CPR) after cardiac arrest (CA) is used to enhance aortic diastolic pressure and, thus, coronary perfusion pressure and blood flow, as well as cerebral blood flow.

Theoretically, together with effective CPR, an optimal vasopressor should ensure adequate oxygen delivery and not increase oxygen demands, which may set tissues in an oxygen deficient state and hazard tissue viability and function. It has been proposed [9] that depletion of myocardial energy stores after 3–4 min of CA can compromise the ability of the heart to resume organized systolic function after defibrillation.

Epinephrine (EP) and AVP have been tested in CA. EP increases coronary perfusion pressure via α-adrenergic mediated vasoconstriction. However EP exerts β_1_-adrenergic cardiac stimulation, which promotes adverse cardiac effects as increased myocardial oxygen consumption, ventricular arrhythmias and post-resuscitation myocardial dysfunction, and its role as the primary drug administered during CPR has been challenged [10]. Beta-adrenergic blockade during CPR in experimental animal studies resulted in improved restoration of spontaneous circulation (ROSC) after ventricular fibrillation (VF), minimized post-resuscitation myocardial dysfunction and improved survival [11].

Thus, AVP, which does not exert adverse cardiac effects as EP, seems advantageous over EP.

However, robust evidence, provided by randomized, controlled trials, in the literature, do not support a beneficial effect of either AVP or EP administration in CA compared to the other or their combination [12,13].

In the present study we estimated the effect of LPS administration in tissues, with regard to tissue oxygenation, utilizing Near Infrared Spectroscopy (NIRS). NIRS is a non-invasive method utilized to assess tissue oxygenation [14]. It has been used to assess microcirculatory derangements after LPS administration [15]. Also, in septic ICU patients NIRS measurements track changes of tissue oxygenation and are related with the severity of sepsis [16]. In a recent study, NIRS was also used in a porcine model of CA, to evaluate peripheral tissue oxygenation at arrest and during CPR [17].

We hypothesized that the viability of tissues in septic animal models, induced by LPS administration, would be compromised after CA and resuscitation efforts, compared with intact animals also suffering CA, due to the impaired aerobic metabolism in the former group, and would result in a decreased chance of ROSC after CPR and Advanced Life Support treatment implementation.

We aimed to investigate ROSC likelihood after CA in an endotoxemic porcine model compared with non-LPS-treated pigs. Also, considering the theoretical advantages of AVP use over catecholamines in endotoxemia/sepsis, we aimed to check the outcome replacing the first dose of EP with AVP during CPR.

## Methods

### Animal preparation

The experimental protocol was approved by the General Directorate of Veterinary Services (permit No 5683/8-1-2008) according to Greek legislation, with regard to ethical and experimental procedures.

Thirty five healthy piglets (Landrace/White Large) of both sexes, aged 12–16 weeks and weighing 25–35 kg were studied. The animals were fasted overnight but had free access to water. All pigs were premedicated with midazolame and atropine (0.1 mg/kg IM) one hour before anesthesia. Anesthesia was induced with an intravenous bolus of propofol (1 mg/kg) and fentanyl (0.05 mg) via the lateral auricular vein. After endotracheal intubation, the pigs were ventilated with a volume-control ventilator (TAEMA Clarys 2000) with FiO_2_ 0.65, tidal volume 10 ml/kg and respiratory rate adjusted for normocapnia. Correct placement of the tracheal tube was ascertained with inflation and auscultation of both lungs. The tracheal tube was secured at the mouth with a tie. To prevent agonal gasping and its possible interactions with pulmonary and hemodynamic variables during cardiac arrest, muscle paralysis was achieved with cis-atracurium (20 mg) after intubation with additional doses as needed.

Anesthesia was maintained with a continuous infusion of midazolame (5 μg/kg/min). Standard lead II electrocardiagraphic (ECG) traces and systemic arterial blood pressure were monitored continuously (FUKUDA DENCHY Datascope expert DS-5300 W). For continuous measurement of arterial oxygenation (SpO_2_), a pulse oxymeter was placed on the tongue of anesthetized animals. Blood gases were measured in arterial blood. Body temperature was controlled with a heating pad aiming for a core temperature of 38°C.

After a surgical plan of anesthesia was obtained, the right external jugular vein, right carotid and left internal jugular vein were isolated by cut down technique and introducer sheath were placed in each. The left external jugular vein was used for fluid and drug administration. Calibrated micromanometer-tipped catheters (Millar) were placed into the ascending aorta and right atrium via the right carotid artery and right external jugular vein, respectively. Access to the heart for open chest cardiac compressions was achieved through a midline sternotomy. Access to abdominal aorta and inferior vena cava (IVC) was achieved through a retroperitoneal approach.

### NIRS measurements

The principles of NIRS function and the vascular occlusion technique (VOT) have been described elsewhere [18,19]. In short, NIRS function is based on the capacity of chromophores in tissues (mainly hemoglobin) to absorb light at the infrared region (680–800 nm), depending on the oxygen saturation of hemoglobin (Hb). Differences in the absorption spectra of hemoglobin make it possible for the NIRS methodology to estimate the percentage of oxygenated hemoglobin over the total hemoglobin in the underlying tissue volume, i.e. the tissue oxygen saturation (StO_2_), as well as an approximation of the total tissue Hb content.

**Figure 1:**
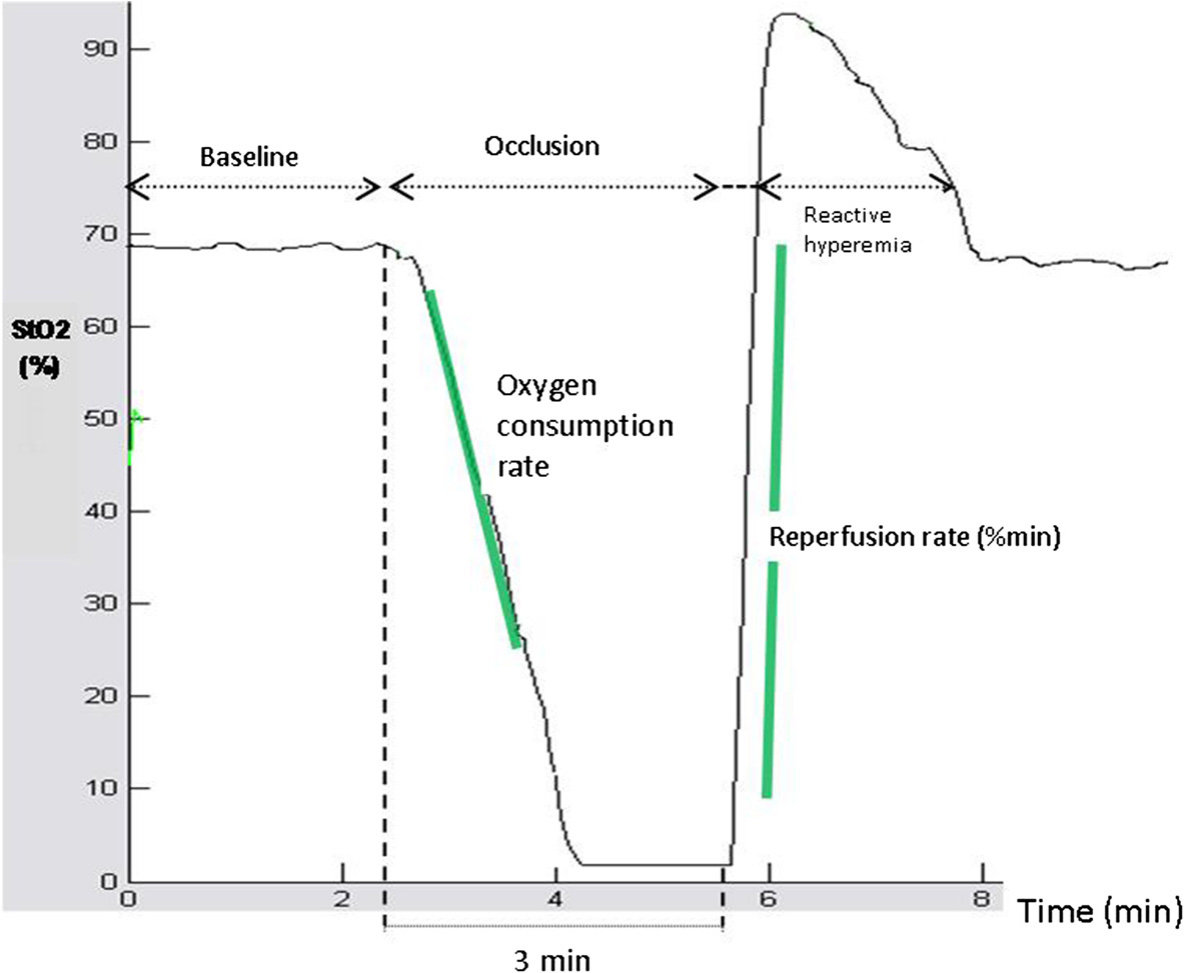
NIRS tracings at baseline and during the vascular occlusion test.

We placed the NIRS probe firmly on a well trimmed and shaved skin area at the medial aspect of the femur of the right rear limb. Underneath are the gracilis, semimembranosis and adductor muscles. This positioning resembles the positioning of the NIRS sensor over the quadriceps femoris muscle in humans, as in a previous study [20]. The light transmitted from the probe has a penetration depth of approximately 25 mm, which enables the measurement of StO_2_ in the corresponding muscle. StO_2_ was measured using the wide-gap, second-derivative NIRS (InSpectra Tissue Spectrometer, Model 325, Hutchinson Technology Inc., Hutchinson, MN, USA). Estimation of tissue oxygenation by NIRS was performed before and after LPS administration. After an initial resting StO_2_ value had been recorded, the VOT test was performed (Figure 1): inferior vena cava and abdominal aorta were accessed by a retroperitoneal approach. Vascular occlusion was performed with a curved forceps and remained for 3 minutes. Then occlusion was rapidly released, producing the reperfusion and the hyperemia phase. StO_2_ was measured continuously during all phases of VOT. Finally, StO_2_ values were again stabilized to a post-VOT resting level.

VOT-derived StO_2_ parameters were analyzed off-line, blindly and in random order, using the InSpectra software (InSpectra Analysis Program, version 2.0; Hutchinson Technology; Hutchinson, MN). The first degree slope of the hemoglobin desaturation curve, during stagnant limb ischemia, reflects the tissue oxygen consumption rate (OCR,%/min). The slope of the increase of StO_2_, after release of the vascular occlusion (reperfusion rate, RR,%/min) and the ratio of StO_2_max to StO_2_min (reactive hyperemia, RH,%) are indicative of endothelial function and vascular reactivity.

### Experimental protocol

**Figure 2:**
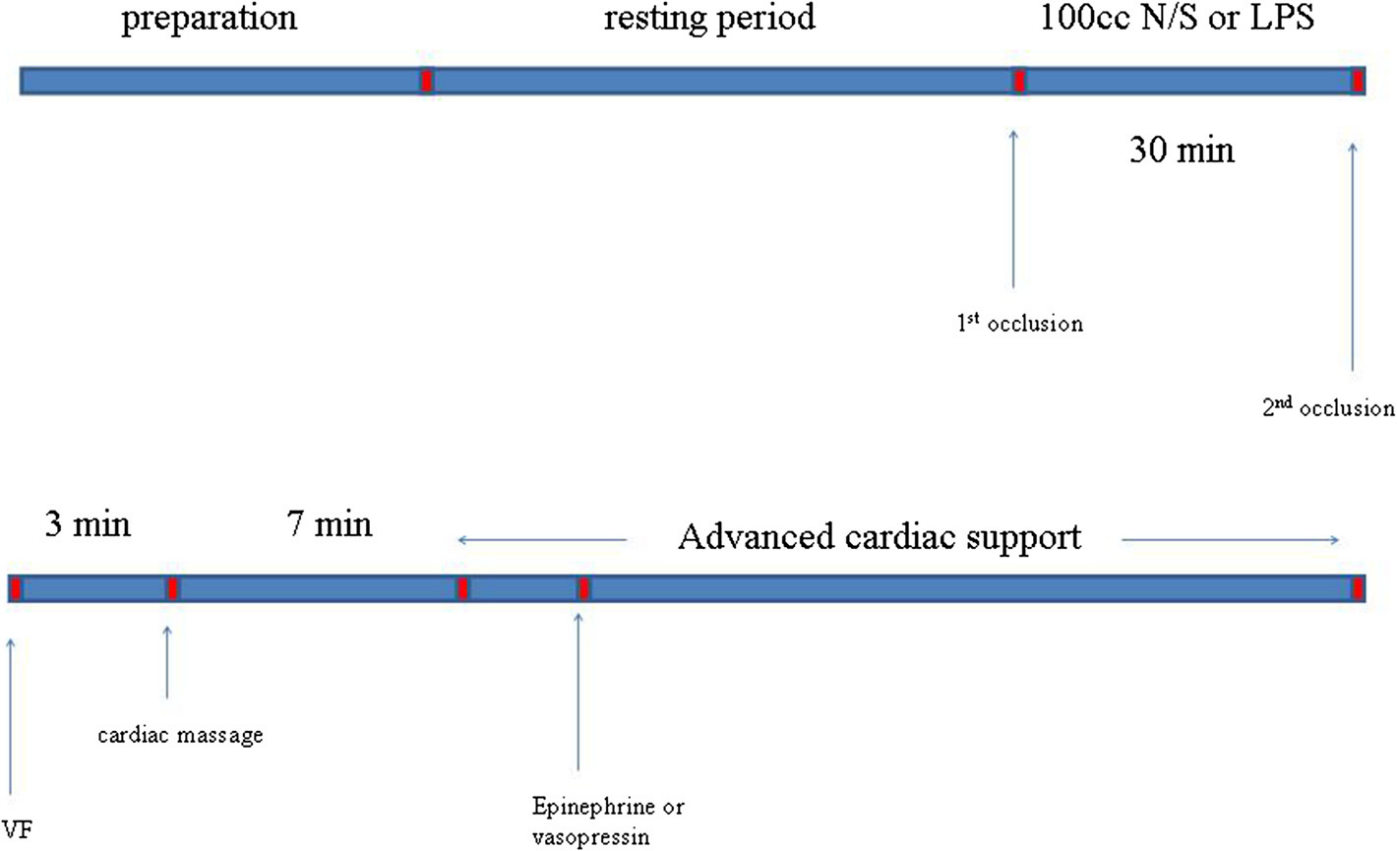
**Timeline of the experimental protocol.** Following surgical preparation and thirty minutes resting period, baseline measurements were performed. Subsequently, 100 ml of normal saline or 100 μg/kg of lipopolysaccharide were administered in a thirty minutes period. New measurements were obtained. Ventricular fibrillation (VF) was induced and mechanical ventilation was discontinued. After three minutes of untreated VF, open chest CPR was performed manually and mechanical ventilation was resumed. Ten minutes after the induction of VF, defibrillation was attempted using 50 joules, followed by two minutes of open chest CPR. Defibrillation was performed again in case of remaining VF. Before the third defibrillation, the animals of group A and B received 1 mg EP whereas the piglets of group C received 0.4 U/kg AVP. The current guidelines for the treatment of cardiac arrest were followed, for the rest of the protocol.

Animals were randomly assigned into three groups: group A (8 animals), group B (14 animals) and group C (13 animals). Figure 2 depicts the timeline of the experimental protocol. Following surgical preparation and thirty minutes resting period, baseline measurements were performed. In order to assess tissue oxygenation we implemented NIRS with VOT in thirteen piglets of the groups B and C (5 animals from group B and 8 from group C). Subsequently, as far as group A is concerned, 100 ml of normal saline were administered in a thirty minutes period. 100 μg/kg of lipopolysaccharide (LPS; 62325 lipopolysaccharide from E.coli serotype O11:B4; Fluca Buchs, Switzerland) was administered in a period of thirty minutes to the piglets of group B and C. New measurements were obtained. NIRS measurements were performed at the piglets that were previously tested. A 50-Hz, 60-V alternating current applied to epicardium was used to induce ventricular fibrillation (VF). VF was confirmed by the typical ECG rhythm and the precipitous decrease in arterial pressure. Then, mechanical ventilation was discontinued.

After three minutes of untreated VF, open chest CPR was performed manually and mechanical ventilation was resumed using identical ventilation variables as before cardiac arrest. The chest compression rate was 100/min with the thumb of the right hand placed on the left ventricle while the fingers encircled the right ventricle. All chest compressions were performed by the same investigator and were metronome guided.

Ten minutes after the induction of VF, defibrillation (NIHON Kohben corporation, TEC-7200 K) was attempted using 50 joules, followed by two minutes of open chest CPR. In case the piglets did not restore an organized cardiac rhythm and circulation, a second defibrillation was attempted followed by two minutes of CPR again.

For groups A and B we used 1 mg of EP before the 3d defibrillation. Subsequently, we used 1 mg of EP every two cycles (every cycle consists of two minutes of CPR) if VF persisted. If not, we used the algorithm of European Resuscitation Council (ERC), regarding the defibrillable and non-defibrillable rhythms.

For group C we used 0.4 U/kg of AVP before the 3d defibrillation. After that we used 1 mg of EP every two cycles (of two minutes of CPR) if VF persisted. If not, we used the algorithm of ERC, regarding the defibrillable and non-defibrillable rhythms.

The vasopressor drugs were infused with a bolus of a crystalloid solution after the administration.

End point for the experiment was ROSC, defined as an organized cardiac rhythm with a mean aortic pressure (MAP) of more than 60 mmHg, lasting for at least five minutes. If spontaneous circulation was not restored 35 minutes after induction of VF, then CPR was stopped (piglets not survived).

### Statistical analysis

Continuous variables were tested for normality of distribution with Shapiro-Wilk test. Pearson’s chi squared test was used for comparisons of categorical variables and paired samples *t*-test or Wilcoxon signed ranks test (in case of not normal distribution) for continuous variables. Independent samples *t*-test or Mann–Whitney signed ranks test were employed to check for between-group differences of continuous variables at baseline. Statistical significance was considered at P < 0.05. Data are expressed as means ± standard deviation or as numbers (percentage). Binary logistic regression analysis was used to investigate differences in ROSC between control (group A) and LPS treated animals (groups B and C). All analyses were conducted using SPSS version 17.0 (SPSS Inc, Chicago, IL, USA).

## Results

Basic characteristics of the piglets are demonstrated in Table 1. None of the piglets regained spontaneous circulation without defibrillation. In Group A, one regained spontaneous circulation after the 1^st^ defibrillation and one after the 2^nd^ defibrillation. In Group B, one regained spontaneous circulation after the 1^st^ defibrillation and one after the 2^nd^ defibrillation. In Group C, one regained spontaneous circulation after the 2^nd^ defibrillation. Vasopressors were administered during CPR in animals that were not resuscitated after two defibrillations.

**Table 1:** Basic characteristics of the piglets in the three groups

**Variables**	**Group A (N = 8)**	**Group B (N = 14)**	**Group C (N = 13)**
Male/female	3/5	8/6	6/7
Weight, kg	27 ± 2	27 ± 2	29 ± 2
HR, bpm	112 ± 26	113 ± 29	97 ± 10
MAP, mmHg	87 ± 13	94 ± 26	89 ± 23
CVP, mmHg	9 ± 5	6 ± 3	7 ± 2
Hct,%	27 ± 7	27 ± 6	27 ± 6
SpO_2_,%	99 ± 1	99 ± 1	99 ± 2
PO_2_, mmHg	334 ± 138	237 ± 85	243 ± 101
PCO_2_, mmHg	63 ± 31	43 ± 21	42 ± 17
pH	7.29 ± 0.16	7.46 ± 0.17†	7.43 ± 0.13*
HCO_3_, mmol/L	28 ± 3	27 ± 3	26 ± 3
K, mmol/L	3.5 ± 0.4	3.6 ± 0.5	3.7 ± 0.5
Na, mmol/L	143 ± 3	144 ± 3	141 ± 5
Ca, mmol/L	1.3 ± 0.4	1.1 ± 0.2	1.1 ± 0.2

Data are means ± SD.

*HR*: heart rate; *MAP*: mean arterial pressure; *CVP*: central venous pressure; Hct: hematocrit; SpO_2_: arterial oxygen saturation; PO_2_: arterial partial pressure of oxygen; PCO_2_: arterial partial pressure of carbon dioxide.

*P < 0.05: comparison between group C and A.

†P < 0.05: comparison between group B and A.

The values (median and minimum-maximum value) of the number of CPR cycles performed in groups A, B and C were 4.5 (2–12), 6.5 (2–12) and 10 (3–12) respectively. Also, the values (median and minimum-maximum value) of the number of EP doses administered during resuscitation in groups A, B and C were 3 (1–4), 3 (1–7) and 4.5 (0–7) respectively.

Survival rates differed significantly (P = 0.046) between the piglets of group A and those of pooled LPS-treated groups, B and C (Table 2). The difference between groups A and C did not reach statistical significance (P = 0.063). No difference was established between groups B and C (P = 0.55).

**Table 2:** Survival rate in the three piglet groups

	**Survived**	**Not survived**	**Sum**	**Survival rate %**
**Group Α**	6	2	8	75
**Group Β**	5	9	14	36
**Group C**	4	9	13	31

After LPS administration MAP and heart rate (HR) did not change (89 ± 22 vs 82 ± 22 mmHg, 105 ± 22 vs 102 ± 26 bpm respectively, P > 0.05) while central venous pressure (CVP) slightly increased (6 ± 2 vs 7 ± 3 mmHg, P = 0.033).

Differences of laboratory variables (such as pH, PCO_2,_ PO_2,_ Hct, Na, K, Ca, HCO_3,_ SpO_2_) of groups B and C, before and after LPS infusion, are shown in Table 3. pH, HCO_3_ and PO_2_ were significantly, although slightly, decreased after LPS administration.

**Table 3:** Comparison of laboratory variables before and after LPS infusion

**Variables**	**Before LPS (N = 27)**	**After LPS (N = 27)**	***P***
pH	7.45 ± 0.15	7.40 ± 0.13	0.04
PO_2_, mmHg	240 ± 93	204 ± 111	0.04
PCO_2_, mmHg	42 ± 19	43 ± 16	ns
HCO_3_, mmHg	26.8 ± 3.2	25.1 ± 3.0	< 0.01
Hct,%	26.9 ± 6.2	27.8 ± 5.7	ns
Na, mmol/L	143 ± 5	143 ± 3	ns
K, mmol/L	3.6 ± 0.5	3.8 ± 0.8	ns
Ca, mmol/L	1.06 ± 0.19	1.16 ± 0.23	0.06
SpO_2_,%	99 ± 1	97 ± 6	ns

Pooled results for both B and C groups. Data are means ± SD.

PO_2_: arterial partial pressure of oxygen; PCO_2_: arterial partial pressure of carbon dioxide; HCO_3_: bicarbonate concentration in blood; Hct: hematocrit; SpO_2_: arterial oxygen saturation.

The comparison between dynamic oxygenation indices obtained by NIRS, before and after the endotoxin infusion, is depicted in Table 4. Although we applied NIRS in thirteen piglets, analysis of the results was not carried out in all animals that where tested, for technical reasons of the analysis procedure. OCR decreased after the septic insult; however the difference did not reach a statistically significant level (P = 0.07).

**Table 4:** NIRS variables before and after administration of LPS

**Variables (N)**	**Before LPS**	**After LPS**	***P***
StO_2_,% (12)	66 ± 14	65 ± 15	0.72
OCR,%/min (12)	30 ± 30	19 ± 7	0.07
RR,%/min (9)	117 ± 66	119 ± 74	0.96
RH,% (10)	15.4 ± 13.4	13.6 ± 13.6	0.73

Pooled results for both B and C groups. Data are means ± SD.

StO_2_: tissue oxygen saturation; *OCR*: oxygen consumption rate estimated by NIRS; *RR*: reperfusion rate; *RH*: reactive hyperemia.

In order to check whether the occlusion of IVC and abdominal aorta for NIRS measurements could affect survival negatively, we compared the LPS-treated piglets that were estimated or not by NIRS. No difference was noted in relation to survival rate (p = 0.18) between the LPS-treated piglets subjected to the VOT procedure (6 out of 13 survived) compared with the ones that were not subjected (3 out of 14 survived).

## Discussion

In the present study the chance of ROSC after CA in an endotoxemic porcine model was reduced compared with non-LPS-treated animals. AVP administration, substituting the first dose of EP during CPR, did not influence the outcome.

The results of studies investigating the effectiveness of AVP (alone or in combination with EP), either in restoring spontaneous circulation after CA or regarding the long-term survival and the neurologic recovery, have been inconsistent.

In a recent randomized study, patients with CA presenting to or in the emergency department, were randomly assigned to receive either 1 mg of EP or 40 U of AVP and additional doses of EP. AVP improved outcomes in patients with prolonged arrest times and seemed to improve survival at admission [21].

However, most randomized, controlled trials failed to prove any clear benefit for either drug administration over the other [12,13] while recent studies question the ability of EP to offer any overall benefit in CA patients [10].

In animal experiments although AVP seemed to be superior to placebo or EP alone [22], improving hemodynamic parameters, coronary perfusion pressure and chance of ROSC [23], other studies have provided contrasting results [24].

Concerning our septic model this is the first study to provide an experimental confirmation of the adverse effect of sepsis on survival after arrest.

In the endotoxic environment created pre-CA, death rate more than doubled. Adequate tissue oxygen delivery and utilization, and avoidance of an untimely ATP depletion seem essential for the heart, to resume an organized rhythm during the advanced resuscitative treatment [9].

The decline of oxygen consumption rate after LPS administration indicates that conditions of impaired aerobic metabolism begin to prevail in peripheral tissues, early after the septic stimulus, before CA. The incipient reduction of pH, HCO_3_ and PO_2_ can be viewed in the same context.

In this septic background, AVP administration did not improve the outcome in comparison to EP alone, as hypothesized. Therefore the efficacy of AVP in endotoxemia and sepsis is questioned although a recent meta-analysis suggests that AVP administration could be beneficial in septic shock [25], possibly due to a relative AVP deficiency observed in septic states [26], in which, nitric oxide (NO) production has a significant role [27,28].

AVP receptor blockade in endotoxic models resulted in lower blood pressure than endotoxin alone [29]. Also, AVP administration inhibited NO production induced by LPS infusion [30] and restored LPS-induced vascular hyporeactivity [31-33].

Although the above considerations can justify some expectations relating to a potential benefit of an, at priority, AVP utilization in this special group of CA patients, the present study did not confirm a positive outcome. Future research, with different study design, dose and timing of AVP usage (e.g. late septic models), is necessary to clarify the issue.

Of note, survival in LPS-treated piglets was not negatively affected by the vascular occlusion procedure. Contrariwise, the piglets subjected to the VOT procedure appeared to have a higher survival rate, which was not statistically significant, likely due to the small sample size.

This can be explained in the context of the ischemia-reperfusion cardioprotective potential, broadly evaluated, since it was shown that short ischemia-reperfusion cycles protect the heart from a subsequent sustained ischemic insult [34]. Interestingly, a recent study in rats demonstrated that limb ischemia preconditioning had a protective effect in myocardial tissue subjected to ischemia for 30 min, followed by reperfusion [35].

## Conclusions

The present study showed that in a septic porcine model of CA, where VF was provoked soon after endotoxin administration, chance of ROSC after resuscitation was significantly reduced in the LPS-treated piglets compared to healthy controls.

AVP administration during CPR, substituting the first dose of EP, did not affect the outcome of septic animals.

### Limitations

Some limitations should be mentioned: 1) In our study we used LPS for the septic model. However intra-abdominal infusion of autofeces is also an accepted model that would be closer to a real clinical situation. 2) Pigs as small as 25–35 kg is the kind we use in our laboratory. Although pigs of this kind have been used in other experimental protocols concerning resuscitation and other pathologies, animals of 60–70 kg would be more comparable to average weighted patients. 3) It would be useful to test AVP in not septic pigs and also to include a sham group. 4) The number of the piglets used was relatively small. 5) Our septic model is limited in terms of severity and duration of sepsis and the results could not be generalized to other cases of more severe and protracted sepsis. 6) There are inherent limitations of the NIRS method to estimate oxygenation indices in peripheral tissue. 7) We did not perform NIRS measurements in all piglets. However, the procedure did not seem to negatively affect survival of septic animals.

### Key messages

•Pre-existing sepsis lowers the chance of ROSC after CA.

•Adding AVP to EP administration, for CA occurring early after a septic insult, does not seem to induce any further benefit.

## Abbreviations

ABG: Air blood gases; CVP: Central venous pressure; CPR: Cardiopulmonary resuscitation; AVP: Arginine-vasopressin; EP: Epinephrine; NE: Norepinephrine; CA: Cardiac arrest; ERC: European Resuscitation Council; VOT: Vascular occlusion technique; Hb: Hemoglobin; Hct: Hematocrit; ECG: Electrocardiography; HR: Heart rate; IVC: Inferior vena cava; LPS: Lipopolysaccharide; MAP: Mean arterial pressure; NIRS: Near Infrared Spectroscopy; OCR: oxygen consumption rate; RR: Reperfusion rate; RH: Reactive hyperemia; ROSC: Restoration of spontaneous circulation; StO_2_: Tissue oxygen saturation; VF: Ventricular fibrillation.

## Competing interests

The authors declare that they have no competing interests.

## Authors’ contributions

All authors have contributed substantially to the submitted work and have read and approved the final manuscript. TL participated in the experimental procedure and collection of data and helped to draft the manuscript. IV drafted the manuscript. HA, VG and EN participated in the experimental procedure and collection of data. EK helped in analysis of the data and statistical analysis. GT participated in the experimental procedure, data acquisition and analysis, helped in statistical analysis and revised critically the manuscript. SN conceived and designed the study and revised the manuscript critically.
